# Therapeutic Drug Monitoring in Oncohematological Patients: A Fast and Accurate HPLC-UV Method for the Quantification of Nilotinib in Human Plasma and Its Clinical Application

**DOI:** 10.3390/biomedicines11030947

**Published:** 2023-03-20

**Authors:** Vanesa Escudero-Ortiz, Francisco José Rodríguez-Lucena, Gabriel Estan-Cerezo, Esther Mancheño-Maciá, Venancio Conesa-García, Ana García-Monsalve, Leticia Soriano-Irigaray, Andrés Navarro-Ruiz

**Affiliations:** 1 Pharmacy and Clinical Nutrition Group, Universidad CEU Cardenal Herrera, 03204 Elche, Alicante, Spain; 2 Pharmacy Service, Hospital General Universitario de Elche-FISABIO, 03203 Elche, Alicante, Spain; 3 Hematology Service, Hospital General Universitario de Elche-FISABIO, 03203 Elche, Alicante, Spain

**Keywords:** therapeutic drug monitoring, nilotinib, HPLC-UV, analitycal method, pharmacokinetic, cancer

## Abstract

Nilotinib, a second-generation tyrosine kinase inhibitor, has demonstrated clinical activity in chronic myeloid leukemia. As an exposure–response relationship has been observed for nilotinib, its therapeutic drug monitoring could be a valuable tool in clinical practice. Therefore, the aim of this study was to develop and validate a selective and precise high performance liquid chromatography–ultraviolet method for the measurement of nilotinib in plasma from patients with cancer. After protein precipitation extraction with acetonitrile, nilotinib and rilpivirine were separated using isocratic elution on a Tracer Excel 120 ODS C18 column using a mobile phase consisting of a mixture of potassium dihydrogen phosphate-buffered solution (pH 5.5; 0.037 M)–methanol–acetonitrile (45:45:10, *v*/*v*/*v*), pumped at a flow rate of 1.7 mL·min^−1^. A wavelength of 254 nm was selected for the quantification of the analyte and the internal standard (IS). The technique was validated following the guidelines for the validation of analytical methods of regulatory agencies (Food and Drug Administration (FDA) and the European Medicines Agency (EMA)). Linearity was established in a concentration range between 125 and 7000 ng/mL. The detection limit was 90 ng/mL, and the lower limit of quantification was 125 ng/mL. For all concentrations in the calibration curve, the intraday and interday coefficients of variation were less than 4.1%. Median recovery of nilotinib from plasma was ≥65.1% (±21.4%). The method described is sensitive, selective, reproducible, and rapid, and can be used for the accurate determination of nilotinib in human plasma for pharmacokinetics studies and for therapeutic drug monitoring (TDM) of nilotinib in routine clinical practice.

## 1. Introduction

Chronic myeloid leukemia (CML) is a myeloproliferative disorder in which an unregulated growth in myeloid cells of the bone marrow occurs followed by accumulation of these cells in the blood. CML is characterized by the presence of the Philadelphia chromosome, which is generated because of the translocation between the long arms of chromosomes 9 and 22. DNA exchange between chromosomes leads to the formation of a new oncogene encoding the BCR-ABL protein that exhibits tyrosine kinase activity [1]. First-line treatment of CML contemplates the use of a tyrosine kinase inhibitor (TKi). Imatinib is a first-generation TKi used in the treatment of CML [2,3]. Although most patients have a good response to treatment with imatinib, clinical resistance may occur in approximately 20% of cases of chronic phase disease and in a higher percentage in later stages. As an alternative to imatinib resistance and for imatinib intolerant patients, a second generation of BCR-ABL TKis have been developed, including nilotinib and dasatinib [4,5,6].

Nilotinib inhibits the tyrosine kinase activity of the BCR-ABL protein. This molecule binds the ATP-binding site of the BCR-ABL protein with higher affinity than imatinib, overriding resistance caused by mutations. It is approved for the treatment of patients with newly diagnosed Philadelphia chromosome-positive (Ph+) CML in a chronic phase (recommended adult oral dose: 300 mg twice daily) and in patients with Ph+ CML in an accelerated phase who are resistant to or intolerant of imatinib (recommended adult oral dose: 400 mg twice daily) [5]. At this dose, the nilotinib relative bioavailability was about 31% [7]. The mean maximum plasma concentration (*C_max_*) 2320 ± 1070 ng/mL was achieved around 3 h (range, 2–8 h) after drug intake, and the mean area under the curve (AUC) was 19.0 ± 9.1 µg·h/mL [8]. Nilotinib should be administered at least 1 h before or 2 h after food intake. The bioavailability of nilotinib increased significantly when the drug was co-administered with a high-fat meal. In relation to that, there has been an increase of up to 112% in the *C_max_* and an increase between 50–82% in the AUC [5]. This drug showed a high interindividual variability in exposure (coefficient of variation, 32–64%), which could be due to its limited bioavailability and its low absorption [9]. The apparent volume of distribution (Vd/F) varies from 393 to 1215 L [10]. In vitro experiments with human plasma indicated that plasma protein binding of nilotinib is approximately 98% and the blood cell/plasma ratio is about 0.7 [5]. Nilotinib is metabolized primarily by cytochrome P450 (CYP) 3A4 isoform and with minor contributions from CYP2C8. After administrating nilotinib, 90% of the dose was recovered in feces unchanged within the following 7 days [5,7].

Like others Tkis, a large interindividual variability on nilotinib pharmacokinetic (PK) has been demonstrated. Causes of this exposure variability occur during ADME processes and may contribute to the variation in the anti-cancer pharmacodynamic effect and to variation in the treatment response [11]. The relationship between nilotinib plasma concentration and treatment efficacy has been studied. Giles et al. [12] described in 493 subjects with CML that patients with a lower trough concentration (*C_min_*) (*C_min_* < 469.4 ng/mL) had significantly longer times to complete the cytogenetic response (*p* = 0.010), longer times for major molecular response (MMR) (*p* = 0.012) and shorter times to progression (*p* = 0.009) compared with patients that show higher *C_min_* values. Moreover, Larson et al. [13] defined that a *C_min_* > 761 ng/mL was significantly associated with a MMR for 12 months on an ROC curve, with a sensitivity of 76.2% and specificity of 77.8%. On the other hand, the relation between the exposure to nilotinib and the treatment toxicity has also been defined. Different studies have shown an important correlation between *C_min_* and AUC values with elevated levels of lipases and bilirubin (*p* = 0.002 and *p* < 0.001, respectively) [12,13]. Correlation between the QT interval prolongation and *C_min_* levels has also been reported [13].

Because maintaining minimal exposure to nilotinib may be relevant in achieving clinical benefit, routine therapeutic drug monitoring (TDM) in clinical practice would reduce the number of patients with suboptimal nilotinib exposure before disease progression. In addition, nilotinib TDM could prevent toxicities that result in the discontinuation of therapy and in the change to another treatment line.

High-performance liquid chromatography coupled with mass spectroscopy (HPLC–MS/MS) is currently among the most sensitive analytical techniques for the quantification of nilotinib in human plasma [14]. This equipment is expensive and is not available in all clinical laboratories to perform nilotinib TDM routinely in clinical practice. As an alternative to this detection technique, some studies have also been published for the quantification of nilotinib in human plasma via high-performance liquid chromatography coupled with ultraviolet detection (HPLC–UV) [15,16,17,18,19,20]. According to Nakahara et al. [20], the use of HPLC–UV is a simple and more economical alternative to HPLC–MS/MS for nilotinib TDM, since there is a strong correlation between nilotinib concentrations determined via HPLC–UV and those determined via HPLC–MS/MS (*r*^2^ = 0.988, *p* < 0.01).

Therefore, one of the objectives of the present study is to monitor nilotinib to individualize the dosing regimen in oncohaematological patients through the estimation of individual pharmacokinetic parameters. To perform this TDM, it is necessary to have an analytical method implemented in routine clinical practice that meets the criteria reported in the guidelines of regulatory agencies for bioanalytical methods, so another objective of this study has been to develop and validate an analytical method that is accurate and precise that allows for the rapid quantification of nilotinib in human plasma.

Compared to other previously published analytical methods using HPLC–UV, the method developed in this study has an advantage in that it has a shorter analysis time than other studies for the determination of nilotinib. Nakahara et al. [20] and Yuki et al. [18], among others, showed total retention times greater than 30 min, and nilotinib peaks were detected at the times of 19.8 min [18] or 25 min [20]. Our method shows shorter retention times, which allows for a better implementation in the daily practice of hospital laboratories.

## 2. Materials and Methods

### 2.1. Chemicals and Reagents

Nilotinib free base >99% (lot BNL-109) was obtained from LC Laboratories (Woburn, MA, USA) and the IS rilpivirine was given by Janssen Global. Chromatography was performed using HPLC-grade acetonitrile, and methanol and water was purchased from J.T. Baker^®^ (Deventer, The Netherlands). Dimethyl sulfoxide (DMSO) and potassium dihydrogen phosphate were obtained from Acofarma (Terrassa, Spain). Different sources of drug-free plasma used for the assessment of the matrix effects and for the preparation of quality controls (QCs) and calibrators were kindly provided by from the Centro de Transfusiones de la Comunidad Valenciana (Valencia, Spain). 

### 2.2. Equipment and Chromatographic Conditions

The equipment used was a HPLC system (Shimadzu, Kyoto, Japan) which consisted of a quaternary pump (model LC-20AD), degasser (model DGU-20AS), autosampler (model SIL-20AC), thermostated column compartment (model CTO-10AS) and a UV-visible detector (model SPD-M20A). Data were acquired and processed with LCsolution^®^ software from Shimadzu Corporation (Kyoto, Japan). Separation of the compounds was achieved by using a Tracer Excel 120 ODS C_18_ column (5 μm; 150 × 4 mm; Teknokroma Analítica SA, Barcelona, Spain) with a guard column packed with the same bonded phase Ultraguard 120 ODS (10 × 3.2 mm; Teknokroma Analítica SA). The chromatography separation was carried out by using a mobile phase consisting of a mixture of potassium dihydrogen phosphate-buffered solution (pH 5.5; 0.037 M)–methanol–acetonitrile (45:45:10, *v*/*v*/*v*), pumped at a constant flow rate of 1.7 mL·min^−1^. Solvents were regularly prepared for each series of analysis. The column was maintained at 35 °C, the injection volume was 20 µL and the eluents were monitored at a wavelength of 254 nm.

### 2.3. Solutions

#### 2.3.1. Preparation of Stock and Working Solutions

Stock solutions of nilotinib (5 mg/mL) and rilpivirine (2 mg/mL) were prepared in DMSO and were stored aliquoted at −80 °C in the dark. Each day of analysis, nilotinib working solutions were prepared freshly by diluting the stock solutions with methanol to give concentrations of 5, 50 and 500 µg/mL. IS working solutions were prepared similarly by diluting in methanol to a final concentration of 20 µg/mL.

#### 2.3.2. Preparation of Calibrators and Quality Control Samples

The calibrators were used to construct a calibration curve consisting of 8 nonzero samples covering the expected range, including the lower limit of quantification (LLOQ). A drug-free blank sample (matrix sample processed without IS) and a zero sample (matrix sample processed with IS) were included according to the recommendations for bioanalytical method validation of the FDA [21] and EMA [22]. Nilotinib calibrators were prepared by diluting working solutions with blank human plasma to obtain concentrations of 125, 250, 700, 900, 2000, 3000, 5000 and 7000 ng/mL. The calibration curve range of 125–7000 ng/mL was selected based on the upper and lower plasma drug levels achievable by the maximum and minimum doses clinically prescribed [7,12,23,24,25]. Furthermore, the QC samples were human plasma samples prepared at known nilotinib concentrations (250, 900 and 5000 ng/mL). Both calibrators and QC were analyzed in the same way as patient plasma samples.

### 2.4. Sample Preparation

All frozen samples were thawed at room temperature before an extraction process. A plasma sample aliquot (500 µL) of the calibrator, QC or patient sample was mixed with a 75 µL of IS working solution at 20 µg/mL in a microcentrifuge polypropylene tube 1.5 mL. The resulting sample was subjected to protein precipitation with acetonitrile (1 mL). The mixture was vortex-mixed for 30 s and centrifuged at 8900× *g* for 20 min at room temperature. The supernatant was transferred into a glass tube and evaporated to dryness under a N_2_ flow at 40 °C. The dry residue was reconstituted with 200 µL of potassium dihydrogen phosphate-buffered solution (pH 5.5; 0.037 M) and vortex-mixed for 30 s until a clear supernatant was obtained. The supernatant previously filtered with nylon filter membranes with a 0.45-µm pore size was transferred into 1.5-mL glass HPLC vials.

### 2.5. Validation Study

Validation of the method was carried out following the recommendations published in the guidelines for the validation of the bioanalytical method of FDA [21] and EMA [22] in terms of linearity, selectivity, accuracy, precision, recovery, and stability.

#### 2.5.1. Linearity and Sensitivity

To estimate the linearity of the method, 3 complete calibration curves (8 concentrations) were analyzed on different days. Linear regression analysis of the data for each calibration curves was performed according to Equation (1):

*y* = *a* + *b* · *C* (1)

where *y* is the peak-height ratio of nilotinib to IS, *C* is the nilotinib concentration and the intercept and slope of the curve are represented as *a* and *b*. Weighting factor for the linear regression was determined according to the result of the model homoscedasticity and the evolution of the variance with respect to the concentration, as has been performed in other studies previously [26]. The slope, intercept and correlation coefficient (*r*) were calculated for each calibration curve. The method was considered linear in the range of concentrations from 125 to 7000 ng/mL if the value of *r* was greater than 0.99 for all calibration curves.

The sensitivity of the method was determined using the detection limit (LOD) and the LLOQ. The LOD was considered as the lower dilution of the LLOQ to give a signal-to-noise ratio higher than 3. The LLOQ was the lower amount of each calibration curve. The LLOQ response of the analyte should be at least 5 times higher than the blank response. To validate the curve, at least 2/3 of the calibrators should be less than 15% of the coefficient of variation (CV), except for the LLOQ, where it was less than 20%.

#### 2.5.2. Selectivity and Specificity

The selectivity of the method was investigated by analyzing the interference of endogenous compounds of human plasma. Blank human plasma from 6 different samples was analyzed with the IS (zero plasma) and without the IS. The interference peak should be less than 5% of the peak area for the LLOQ for both the analyte and IS in plasma. Furthermore, possible interference of co-medication in 6 cancer patients treated with other drugs were evaluated.

#### 2.5.3. Accuracy and Precision

Intraday and interday accuracy and precision were determined by assaying spiked plasma at the LLOQ and in QC samples at 3 different concentrations, low quality control (LQC): 250 ng/mL, medium quality control (MQC): 900 ng/mL and high quality control (HQC): 5000 ng/mL, measuring 5 replicates per concentration on 3 different days. The accuracy was expressed using the mean relative error (MRE) and calculated as: 

$Accuracy=\left[(C_{t}-{\bar{C}}_{obs})/C_{t}\right]\times 100$, where *C_t_* represents the nominal concentration and ${\bar{C}}_{obs}$ the mean of the observed concentration (*C_obs_*). The precision was expressed as the relative standard deviation (RSD) of the different measurements for each nominal concentration using the equation $Precision=\left(SD_{C_{obs}}/{\bar{C}}_{obs}\right)\times 100$, where *SD_Cobs_* represents the standard deviation of *C_obs_* and ${\bar{C}}_{obs}$ the mean of *C_obs_*. The analytical series were considered valid and accepted if for each concentration both MRE and RSD should be lower than ±15%, except for the LLOQ, where they should be less than ±20%.

#### 2.5.4. Recovery

Nilotinib recovery was evaluated at low, medium and high concentrations (250, 900 and 5000 ng/mL). Recovery was performed in all QCs and 5 replicates were used in each case. The recovery parameter after extraction was determined by comparing the peak area of the analyzed concentration in the mobile phase, which represented 100% recovery, with the peak area after extraction in plasma. To consider the adequate recovery, both for nilotinib and for the IS, the values obtained must be precise and reproducible for all the concentrations evaluated.

#### 2.5.5. Stability

The stability of nilotinib at different conditions has already been evaluated in many articles. However, in the present study it has been conducted to study the stability of the stock and working solutions, freeze–thaw stability and autosampler stability. For all stability studies, the analyte stability should fall within 85−115% of the nominal concentration and was not allowed to deviate more than ±15% from the nominal concentration of all the QCs studied, as indicated in the Guidelines for Bioanalytical Methods Validation [22].

The stability of both nilotinib and IS stock solutions was determined after storage in dark conditions at −80 °C for a period of 10 days. On the other hand, the stability of the working solutions in methanol on each test day was evaluated after 8 h at room temperature with light and in the dark.

The stability of nilotinib after freeze–thaw processes was determined by analyzing the QCs during 2 freeze–thaw cycles. 3 aliquots were made for each concentration, which were stored at −80 °C and thawed at room temperature in each cycle.

The stability in the autosampler of the sample after the extraction process was evaluated at room temperature in the range between 2 and 8 h.

### 2.6. Clinical Application of Validate Method

The analytical method validate in the present study has been used, in routine clinical practice, to perform TDM in patients with CML under treatment with nilotinib. The analytical method described above was used to quantify the steady-state drug concentration of plasma samples from the 4 patients included in the study. The TDM process in the patients was carried out after 14 days from the beginning of the treatment since it is the time necessary for the drug to reach the steady state. Doses received by patients were between 200 and 300 mg and were received twice-daily. Blood samples were obtained at the following sampling times: before drug administration and 1, 2 and 4 h after drug administration. Samples collected in lithium heparin tubes were centrifuged at 1000× *g* for 10 min at room temperature. In all cases, the supernatant plasma was separated and stored at −80 °C. 

The study protocol complies with ethical research principles and was reviewed and approved by the local biomedical research ethics committee. For the inclusion of the patients in the PK study, they were previously informed verbally and in writing of the objectives of the study, the benefits that they could have and the risks.

In the PK analysis, some parameters such as the *C_max_* and *C_min_* and the time to reach *C_max_* (*T_max_*) were directly obtained from the plasma concentration–time profile. The truncated AUC_0–12_ was calculated using the linear trapezoidal method. The estimation of the rest of the PK parameters (terminal elimination rate constant (λz), terminal half-life (*t*_1/2_) and apparent oral clearance of nilotinib (dose/AUC_0–12_) was carried out through a monocompartmental analysis using Phoenix WinNonlin Professional 8.3 (Certara France Sarl). 

## 3. Results

### 3.1. Method Development

**Figure 1:**
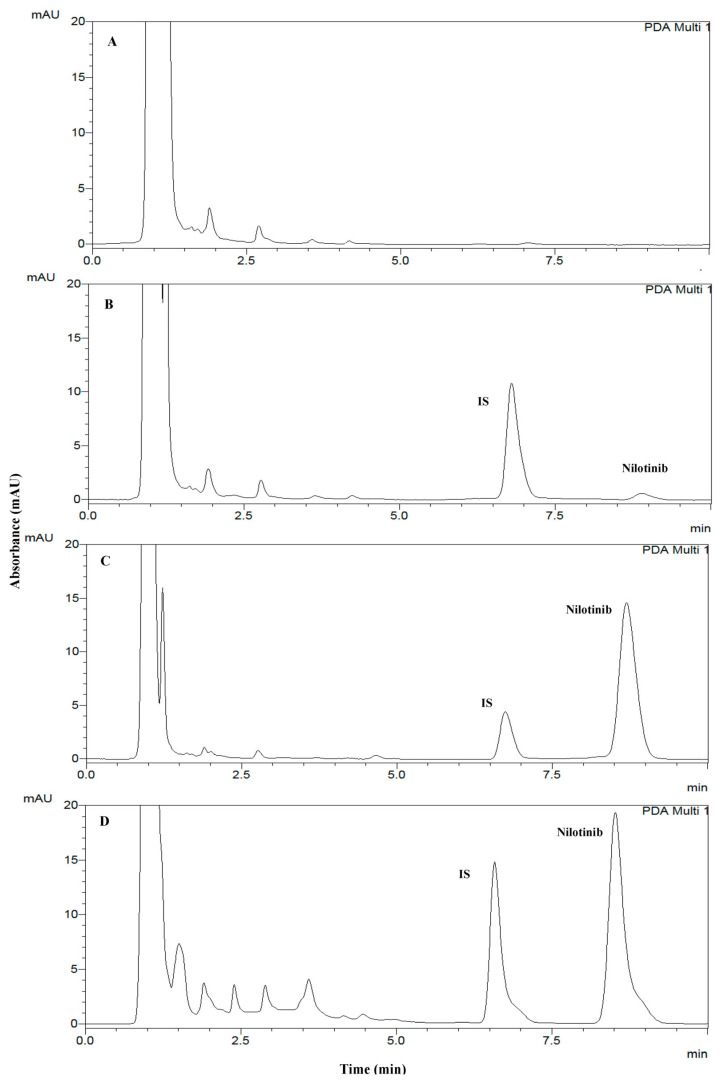
Representative chromatograms: (**A**) blank human plasma without nilotinib, (**B**) blank human plasma at LLOQ (125 ng/mL), (**C**) blank human plasma after addition of 7000 ng/mL of nilotinib and (**D**) a plasma simple from a patient with cancer with 300 mg of nilotinib twice a day. mAU, milliabsorbance units.

Nilotinib is a strong base with a pKa value of 12.38. It has good solubility in DMSO, organic solvents (methanol and acetonitrile) and acidic aqueous solution. Its aqueous solubility decreases significantly with increased pH. Based on its physicochemical properties, a reversed-phase liquid chromatography method was optimized for the separation of nilotinib. This method was employed with a stationary-phase Tracer Excel 120 ODS C18 column (5 μm; 150 × 4 mm; Teknokroma Analítica SA). The retention times for nilotinib and rilpivirine were approximately 8.8 and 6.7 min, respectively. The total run time for each simple analysis was 10 min. Typical chromatograms were obtained with extracted drug free human plasma, samples of plasma spiked with nilotinib (125 ng/mL and 7000 ng/mL) and rilpivirine, and plasmas from one patient treated with nilotinib (300 mg daily) and spiked with IS were clean and without interferences (Figure 1). The assay was specific and selective because no interference was observed with other compounds in 6 plasma samples from 6 cancer patients that were in pharmacological treatment with other drugs usually employed in chronic patients (diuretics, antidepressants, anticholinergic, antihistamines or anesthetics).

### 3.2. Method Validation

#### 3.2.1. Linearity and Sensitivity

The plasma linear calibration curves were constructed using the peak area ratio (*y*) over a nilotinib concentration range of 125 to 7000 ng/mL in human plasma. In the linearity test, a significant difference (*p* < 0.05) was evidenced between the variances for each concentration analyzed in the calibration curve. The weighting factor 1/*y* was used because the variance increased proportionally to the concentration [27]. The 3 calibration curves were linear over a concentration range of 125 to 7000 ng/mL. Calibration parameters are shown in Table 1 and *r* was greater than 0.99 on the 3 validation days.

The determined LLOQ and LOD were 1250 ng/mL and 90 ng/mL, respectively. In the case of the LLOQ, all the samples evaluated (100%) presented homogeneous CVs that did not deviate more than the 20% allowed. For the rest of the concentrations evaluated, 95% of them showed CVs below the 15% allowed (no more details of this data are shown).

#### 3.2.2. Accuracy and Precision

The intraday and interday accuracy and precision of the HQC, MQC and LQC samples are reported in Table 2. For the 3 QC concentrations, intra-assay and inter-assay accuracies range from 3.7% to 10.2% and from 1.8% to 4.2%, respectively. The RSD for the intra-assay and inter-assay results was lower than 4% and 3.9%, respectively. Results of MRE and RSD for the LLOQ were lower than 3.7%.

#### 3.2.3. Recovery

The absolute recoveries of nilotinib from plasma at concentrations of 250, 900 and 5000 ng/mL were 81.0% (±14.7%), 74.0% (±4.9%) and 65.1% (±21.4%), respectively. The absolute recovery of the IS was 67.0% (±21.4%) at the used concentration.

#### 3.2.4. Stability

The stock solutions of nilotinib and rilpivirine stored for 10 days at −80 ºC were comparable with the freshly made ones; the value for accuracy (CV for the replicates, %) obtained was less than 1.5% (3.7%) for both drugs. Working solutions of nilotinib and rilpivirine were stable for at least 8 h at room temperature, with or without light (the value for accuracy (CV, %) obtained was lower than 15.1% (6.5%) for both drugs). 

Nilotinib was also stable for up to 8 h on the autosampler without any significant degradation (<8.3%). The main results of additional stability studies performed for plasma samples, expressed as accuracy values, are shown in Table 3. Results show that the stability of the analytes is acceptable under all stability tests. Data for 2, 4 and 6 h of short-term stability with and without light are not shown in Table 3.

### 3.3. Clinical Application of Validate Method

**Figure 2:**
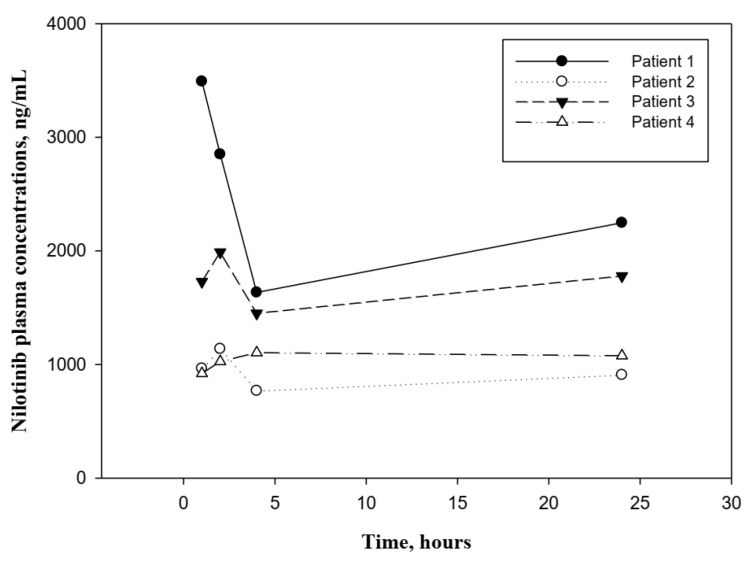
Plasma concentrations profile of nilotinib after administration of the drug to 4 patients receiving nilotinib. The symbols represent the plasma concentrations of nilotinib of patients at different times. The lines represent the union of the different values of plasma nilotinib concentrations for each patient.

To show the applicability of the method in routine clinical practice, 16 plasma samples from 4 patients with CML in treatment with nilotinib were analyzed. Figure 2 shows the plasma concentrations profiles of nilotinib in these patients, and the corresponding pharmacokinetics parameters are summarized in Table 4.

The comparison analysis of nilotinib exposure in the present study, versus the exposure values described in the scientific literature as therapeutic efficacy targets, shows that 100% of treated patients have *C_min_* values higher than the therapeutic target of efficacy (*C_min_* ≥ 761 ng/mL [13]). Figure 2 shows that all concentrations in the 4 concentration–time profiles are higher than the therapeutic target described in the literature.

To explore the effect of drug exposure on the adverse event occurrence, an analysis of the relationship between drug exposure (*C_min_* and *C_max_*) and the toxicity variables shown in Table 5 has been performed. A moderate–high correlation (*r* > 0.8) is observed between the values of the exposure variables (*C_min_* and *C_max_*), with the appearance of hematological toxicity (leukocytes count).

## 4. Discussion

TDM in oncohematological patients is implemented with the aim of individualizing therapy to achieve greater efficacy and reduce possible toxicity associated with treatment. In order to implement the TDM tool in routine clinical practice, it is necessary to have analytical methods that allow the quantification of the drugs, as well as PK models that allow for the estimation of PK parameters for each patient. In this regard, the present study developed and validated an analytical method for the quantification of nilotinib concentration in human plasma. The measurement of nilotinib plasma concentrations, in fact, was useful to evaluate patient adherence to daily oral therapy, potential drugs interactions, treatment efficacy and severe drug related adverse events.

In the analytical method optimization phase, different conditions were tested in terms of the stationary phase and mobile phase. The mixtures of the potassium dihydrogen phosphate buffer or the ammonium acetate buffer and acetonitrile or methanol were evaluated to achieve the best chromatographic peak shape and resolution for nilotinib and rilpivirine with a minimum run time. Finally, the mixture of potassium dihydrogen phosphate-buffered solution (pH 5.5; 0.037 M), methanol and acetonitrile (45:45:10, *v*/*v*/*v*), pumped at a constant flow rate of 1.7 mL·min^−1^, produced optimal separation with very sharp and symmetrical peak shapes for both IS and nilotinib, as reported elsewhere [15,16,17,18,19]. The total run time for each sample analysis was 10 min, which is 71% shorter than the run time previously reported for another HPLC–UV assay [16], and similar to other previous studies of the same characteristics [19]. The run time of 10 min is an optimal time for the implementation of the method in routine clinical practice.

The assay used acetonitrile for sample protein precipitation and extraction of the analyte and IS. The absolute recoveries of nilotinib from plasma reported by the authors were higher than 65%. These recovery data are considered adequate and sufficient for the quantification of nilotinib in the plasma samples of the patients treated at the doses defined in the present study.

Guaranteeing the stability of the solutions is a very important aspect to consider during the development and validation process of the analysis method. The stability studies carried out in the present work show how stable nilotinib and rilpivirine were under the conditions evaluated. All the results obtained were comparable to those previously published in the literature [28,29]. 

Regarding the sensitivity of the analytical method, on the one hand the LLOQ has been like that established by other authors [16], while on the other hand it is 25 times higher than the LLOQ published in other studies with HPLC–UV [15,17,19]. However, some clinical trials reported that *C_min_* at a steady state in patients receiving 300 mg twice-daily was close to 1123 ng/mL, which is higher than the LLOQ of the method described in the present study. As the dose range of nilotinib administration in the present study is 200–300 mg twice-daily, the assay LLOQ (125 ng/mL) is deemed adequate to monitor nilotinib concentrations in patients with cancer.

Pharmacokinetic parameters of nilotinib determined in 4 patients with CML in this study are like those previously reported for patients with the same pathology treated with 300 mg and 400 mg twice-daily [13]. The mean *C_min_* and *C_max_* of 4 patients are 1193.5 ng/mL and 1930.1 ng/mL, similar values to the ones determined by Larson et al. in 17 patients treated with nilotinib 300 mg twice-daily (1123 ng/mL and 1360 ng/mL, respectively) [13]. Additionally, the mean steady-state AUC_0–12_ reported for these 17 patients was 11.9 ng·h/L, whereas the mean value reported in this study is 16.4 ng·h/L.

## 5. Conclusions

In the present study, an analytical method has been reported for the determination of nilotinib in a single chromatography run. The HPLC–UV method described here is sensitive, selective, reproducible and rapid, and can be used for the accurate determination of nilotinib in human plasma for pharmacokinetics studies and for TDM. UV detection provides the required level of sensitivity for measuring a pharmacologically relevant concentration of nilotinib in cancer patients when treating with a clinical standard dose, and it could be successfully implemented in routine clinical practice for nilotinib TDM.

## Figures and Tables

**Table 1:** Calibration curves parameters.

	Linearity Equation, *y = a + b · C*		
Day	*a* (×10^−3^)	*b* (×10^−3^)	*r*
1	−6.7	0.7	0.996
2	−8.3	0.7	0.997
3	−5.3	0.7	0.998

**Table 2:** Accuracy and precision of nilotinib determination in human plasma.

Theoretical Concentration, (ng/mL)	Intraday *			Interday *		
	Mean Observed Concentration (SD), (ng/mL)	Accuracy (MRE, %)	Precision (RSD, %)	Mean Observed Concentration (SD), (ng/mL)	Accuracy (MRE, %)	Precision (RSD, %)
125	120.3 (4.1)	3.7	3.4	121.6 (3.2)	2.7	2.6
250	275.6 (11.1)	10.2	4.0	267.5 (10.5)	7.0	3.9
900	866.9 (12.3)	3.7	1.4	883.3 (18.4)	1.8	2.1
5000	4705.8 (96.7)	5.9	2.1	4791.4 (147.6)	4.2	3.1

* Results expressed as mean (SD) from 5 replicates.

**Table 3:** Nilotinib stability in human plasma.

Theoretical Concentration, (ng/mL)	Freeze–Thaw Stability *		Short-Term Stability with Light *	Short-Term Stability without Light *
	Cycle 1	Cycle 2	8 h	8 h
250	−11.6 (1.2)	−9.3 (1.0)	0.3 (13.2)	14.1 (1.2)
900	−14.7 (3.8)	−116. (3.1)	15.0 (1.6)	2.8 (4.2)
5000	−14.2 (2.0)	−13.5 (2.5)	−2.6 (3.3)	−15.0 (6.2)

* Results expressed as accuracy value (coefficient of variation of replicates, CV, %).

**Table 4:** Pharmacokinetics parameters of nilotinib.

Pharmacokinetics Parameters	Patient 1	Patient 2	Patient 3	Patient 4	Mean (SD)	CV (%)
*C_max_* (ng/mL)	3491.4	1137.2	1988.4	1103.5	1930.1 (1118.5)	58
*C_min_* (ng/mL)	1633.9	768.9	1450.3	920.7	1193.5 (414.7)	35
*T_max_* (h)	1	2	2	4	2.3 (1.3)	56
AUC_0–12_ (ng·h/L)	24.7	10.1	18.9	11.8	16.4 (6.7)	42
CL/F (L/h)	2.8	1.9	0.6	5.7	2.8 (2.2)	77
*t*_1/2_ (h)	25.1	69.3	187.4	30.7	78.1 (75.4)	96

*C_max_*, peak plasma concentration; *C_min_*, trough plasma concentration; *T_max_*, time to reach *C_max_*; *t*_1/2_, elimination half-life; AUC_0–12_, area under the serum concentration–time curve from 0–12 h; CL/F, apparent oral clearance; CV, coefficient of variation.

**Table 5:** Correlation between exposure to nilotinib and developed toxicity.

	*C_min_*	*C_max_*
Leukocytes (×10^3^/µL)	−0.9194	−0.8439
Neutrophils (×10^3^/µL)	−0.0280	0.1043
Platelet (×10^3^/µL)	−0.6949	−0.5802
Haematocrit (%)	0.0870	0.0096
Haemaglobin (g/dL)	−0.0319	−0.0818
Serum bilirrubin (mg/dL)	−0.4013	−0.3427
Alkaline phosphatase (U/L)	0.7497	0.7101
AST (U/L)	−0.0833	−0.2038
ALT (U/L)	−0.3552	−0.4533
Serum creatinine (g/dL)	−0.1897	−0.3482

*C_max_*, peak plasma concentration; *C_min_*, trough plasma concentration.

## Data Availability

The datasets generated during the current study are available from the corresponding author upon reasonable request.
